# Estrogen receptor and HER2/neu status affect epigenetic differences of tumor-related genes in primary breast tumors

**DOI:** 10.1186/bcr2098

**Published:** 2008-05-16

**Authors:** Eiji Sunami, Masaru Shinozaki, Myung-Shin Sim, Sandy L Nguyen, Anh-Thu Vu, Armando E Giuliano, Dave SB Hoon

**Affiliations:** 1 Department of Molecular Oncology, The John Wayne Cancer Institute, Saint John's Health Center, Santa Monica Blvd, Santa Monica, California 90404, USA; 2 Department of Biostatistics, The John Wayne Cancer Institute, Saint John's Health Center, Santa Monica Blvd, Santa Monica, California 90404, USA; 3 Department of Surgical Oncology, The John Wayne Cancer Institute, Saint John's Health Center, Santa Monica Blvd, Santa Monica, California 90404, USA

## Abstract

**Introduction:**

Estrogen receptor (ER)-positive breast cancers are considered prognostically more favorable than ER-negative tumors, whereas human epidermal growth factor receptor (HER)2/neu-positive breast cancers are associated with worse prognosis. The objective of the present study was to determine whether ER-positive and ER-negative status relates to epigenetic changes in breast cancer-related genes. To evaluate epigenetic differences in tumor-related genes relating to ER and HER2/neu status of primary tumors, we examined the promoter methylation status of the promoter region CpG islands of eight major breast tumor-related genes (*RASSF1A*, *CCND2*, *GSPT1*, *TWIST*, *APC*, *NES1*, *RARβ2*, and *CDH1*).

**Methods:**

Paired ER-positive (*n* = 65) and ER-negative (*n *= 65) primary breast tumors (*n* = 130) matched for prognostic factors were assessed. DNA was extracted from paraffin-embedded tumor tissue after microdissection, and methylation-specific PCR and capillary-array electrophoresis analysis were performed.

**Results:**

In early stages of tumor progression (T1 and N0), *RASSF1A *and *CCND2 *were significantly (*P *< 0.05) more methylated in ER-positive than in ER-negative tumors. *GSTP1 *hypermethylation was more frequent in the lymph node metastasis positive group than in the negative group. Double negative (ER-negative, HER2/neu-negative) breast cancers had significantly lesser frequencies of *RASSF1A*, *GSTP1*, and *APC *methylation (*P *< 0.0001, *P *< 0.0001, and *P *= 0.0035, respectively). Both ER and HER2/neu status correlated independently with these epigenetic alterations.

**Conclusion:**

We demonstrated significant differences in tumor-related gene methylation patterns relevant to ER and HER2/neu status of breast tumors. This may be of significance in the assessment of targeted therapy resistance related to ER and HER2/neu status in breast cancer patients.

## Introduction

Hypermethylation is an epigenetic change that blocks the promoter region of a gene and results in gene silencing. In breast cancer, tumor-related genes may be silenced by hypermethylation; many hypermethylated genes have been reported, and silencing of these genes plays important roles in carcinogenesis and tumor progression [1-3]. Identification of epigenetic changes and their correlation with other clinical factors could lead to improvements in cancer diagnosis and treatment.

In patients with breast cancer, estrogen receptor (ER) status is an important treatment and prognostic factor. Breast cancer patients with ER-positive tumors generally have a more favorable prognosis than do those who have ER-negative tumors. These breast cancer patients can be treated successfully with hormonal therapies such as tamoxifen and aromatase inhibitors [4-9]. Epidemiological studies revealed that patients with ER-positive tumors have risk profiles different from patients with ER-negative tumors. Parity and timing of births were inversely associated with ER-positive tumors, but not with ER-negative tumors, and body mass index after menopause was more strongly associated with ER-positive than with ER-negative tumors [10]. In addition, rates of ER-positive breast cancer incidence have been shown to increase after age 50 to 54 years, whereas the rates of ER-negative breast cancer incidence do not [11].

Similarly, differences in the gene expression patterns of ER-positive and ER-negative tumors have been documented in microarray expression studies, which identified profile differences in breast tumor subtypes [12,13]. These findings suggest that ER status of breast cancer represents distinct phenotypes. However, few studies have determined epigenetic changes in tumor-related genes in relation to ER status in matched-paired breast cancers.

To investigate the epigenetic differences between ER-positive and ER-negative breast cancer, we assessed the methylation frequency of several breast tumor-related genes that are known to undergo hypermethylated changes in breast cancer, and that play important roles in tumorigenesis and cancer progression. The objective of the study was to determine the association between ER status and epigenetic changes in these tumor-related genes.

The genes assessed were as follows: *RASSF1A *(RAS association domain family 1A; location: 3p21.3; GenBank: AF132675), *CCND2 *(cyclin D2; location: 12p13; GenBank: AF518005), *GSTP1 *(glutathione S-transferase P1; location: 11q13; GenBank: U12472), *TWIST *(human basic helix-loop-helix DNA binding protein; location: 7p21.2; GenBank: U80998), *APC *(adenomatous polyposis coli; location: 5q21-q22; GenBank: M74088), *NES1 *(normal epithelial cell-specific 1 or kallikrein 10; location: 19q13.3-q13.4; GenBank: AF024605), *RARβ2 *(retinoic acid receptor-β2; location: 3p24; GenBank: X07282), and CDH1 (E-cadherin; GenBank: L08599). *RASSF1A *is a putative tumor-suppressor gene that is frequently inactivated epigenetically rather than in a mutational event [14]. A direct correlation between promoter region methylation and loss of expression has been shown in many tumor cell lines, including breast cancer [15-18]. RASSF1A can exert a tumor-suppressing effect by blocking oncogene-mediated c-Jun amino-terminal kinase activation [19]. It also associates with microtubules and contributes to the maintenance of genomic stability [20]. Loss of CCND2 expression caused by methylation is an early event in breast cancer tumorigenesis [21]. Methylation of *CCND2 *has been correlated with poor prognosis, implying that CCND2 has a tumor-suppressor function [22]. GSTP1 is one of a family of enzymes that detoxifies hydrophobic electrophiles, and may be part of a protection system from environmental or dietary carcinogens [23]. Our group has previously found that *GSTP1 *methylation correlates with increased tumor size and increased likelihood of sentinel lymph node metastases [24]. TWIST induces E-cadherin mediated cell-cell adhesion and induction of cell motility. Increased expression of *TWIST *correlates with tumor invasion and metastasis [25,26]. APC gene germline mutations have been shown to be associated with familial adenomatous polyposis. Hypermethylation of the *APC *promoter is also associated with breast cancer, especially lobular breast cancer [27-29]. *NES1 *is a putative tumor suppressor gene found to be downregulated in breast cancer [30,31]. *RARβ2 *is postulated to be a tumor suppressor gene. *RARβ2 *methylation correlates with breast cancer metastasis [32]. It is through retinoic acid receptors that retinoids can prevent primary tumor progression [33]. CDH1 expression reduction is regarded as one of the main molecular events involved in dysfunction of the cell-cell adhesion system, triggering cancer invasion and metastasis. Mahler-Araujo and coworkers [34] reported a correlation between negative or reduced CDH1 expression and lack of ER expression in tumors from 245 breast cancer patients.

This study was conducted to investigate epigenetic differences in specific tumor-related genes between ER-positive and ER-negative breast cancers. We hypothesized that ER-positive breast tumors have different epigenetic profiles of tumor-related genes during early stages of cancer progression. We examined the methylation status of eight genes suspected of being involved in regulation of breast cancer, and investigated the methylation status of those genes at different stages of tumor development. We compared the methylation status of these genes between ER-positive and ER-negative breast tumors in early and advanced stages to investigate whether epigenetic changes occur in early stages of primary tumor progression.

Human epidermal growth factor receptor (HER)2/neu is an important factor for treatment and prognosis. HER2/neu over-expression occurs in 15% to 25% of breast tumors and is associated with poor prognosis and resistance to hormonal therapy [35-37]. HER2/neu and ER expression have been reported to exhibit an inverse correlation [9,38,39]. Furthermore, previous reports have demonstrated the effect of estrogen on downregulation of HER2/neu production [40]. We investigated the epigenetic differences between HER2/neu-positive and HER2/neu-negative breast tumors relative to ER status and further identified the epigenetic characteristics of ER-negative, HER2/neu-negative (double-negative) breast tumors.

## Materials and methods

### Tumor and patient selection

Sixty-five paraffin-embedded, invasive ER-negative and ER-positive breast tumor pairs were matched for patient age, tumor size (T stage), nodal status (lymph node [LN] metastasis positive or negative), and presence or absence of distant metastases (M status). The primary tumor characteristics are listed in Tables 1 and 2. HER2/neu status was originally scored 0 to 3+, and 0 and 1+ tumors were regarded as HER2/neu negative. Tumors were graded and staged according to the American Joint Committee on Cancer 6th Edition Guidelines [41]. Informed consent for use of all human specimens in this study was obtained under a protocol approved by Saint John's Health Center (Santa Monica, CA, USA)/John Wayne Cancer Institute institutional review board.

**Table 1:** Patient and tumor characteristics

Clinical factors	ER negative (*n *= 65)	ER positive (*n* = 65)
Menopausal status		
Premenopause	35	35
Postmenopause	30	30
T stage		
T1c	24	24
T2	37	37
T3	4	4
N stage		
N0	38	38
N1	26	27
N2	1	0
M stage		
M0	65	65
M1	0	0
AJCC stage		
I	17	17
IIa	28	28
IIb	16	16
IIIa	4	4
IV	0	0
HER2/neu	14/51 (27%)	15/56 (27%)

Total	65	65

ER, estrogen receptor; HER, human epidermal growth factor receptor.

**Table 2:** Clinical factors by HER2/neu status

Clinical factors	HER2/neu negative	HER2/neu positive	*P *value^a^
T stage			
T1	29	10	
T2/3	49	16	NS
N stage			
N0	50	15	
N1	28	14	NS
Age (years)	50.2 ± 11.3	50.8 ± 12.6	NS

^a^χ^2 ^test and Student's *t*-test. HER, human epidermal growth factor receptor; NS, not significant.

### DNA processing and methylation-specific PCR

DNA was extracted as previously described [24]. Briefly, paraffin-embedded primary tumor specimen blocks were sectioned at 10 μm and deparaffinized in 100% xylene, followed by a 100% ethanol incubation, and stained with hematoxylin and eosin. Tumor tissue was microdissected in comparison with a similarly stained and cover-slipped reference slide cut from each tissue block. DNA preparation buffer containing 50 mmol/l Tris, 1 mmol/l EDTA, 2.5% Tween-20, and Proteinase K (QIAGEN, Valencia, CA, USA) was added to microdissected tissue and incubated at 50°C overnight. DNA was extracted from the aqueous layer using phenol-chloroform-isoamyl alcohol (25:24:1; Fisher Scientific, Pittsburgh, PA, USA) and precipitated using pellet paint NF co-precipitant (Novagen, Madison, WI, USA). DNA was resuspended in molecular biology grade H_2_O (Fisher Scientific). DNA quantification was performed on all specimens using the PicoGreen quantification assay (Molecular Probes, Eugene, OR, USA). Bisulfite modification was performed as previously described [15,24]. A panel of eight genes was assessed for methylation status: *RASSF1A*, *APC*, *TWIST*, *CDH1*, *GSTP1*, *NES1*, *CCND2*, and *RARβ2*. Methylation-specific PCR (MSP) was performed using Ampli*Taq *Gold DNA polymerase (Perkin Elmer, Norwalk, CT, USA) and 50 pmol each of forward and reverse primers for methylated (M) and unmethylated (U) sequences. Primer sequences for MSP of the eight genes are shown in Table 3.

**Table 3:** Primer sequences for MSP

Gene	Methylated/unmethylated	Direction	Sequence
*RASSF1A*	Methylated	Forward	5'-GTGTTAACGCGTTGCGTATC-3'
		Reverse	5'-AACCCCGCGAACTAAAAACGA-3'
	Unmethylated	Forward	5'-TTTGGTTGGAGTGTGTTAATGTG-3'
		Reverse	5'-CAAACCCCACAAACTAAAAACAA-3'
*CCND2*	Methylated	Forward	5'-TACGTGTTAGGGTCGATCG-3'
		Reverse	5'-CGAAAACATAAAACCTCCACG-3'
	Unmethylated	Forward	5'-GTTATGTTATGTTTGTTGTATG-3'
		Reverse	5'-TAAAATCCACCAACACAATCA-3'
*GSTP1*	Methylated	Forward	5'-TTCGGGGTGTAGCGGTCGTC-3'
		Reverse	5'-GCCCCAATACTAAATCACGACG-3'
	Unmethylated	Forward	5'-GATGTTTGGGGTGTAGTGGTTGTT-3'
		Reverse	5'-CCACCCCAATACTAAATCACAACA-3'
*APC*	Methylated	Forward	5'-TATTGCGGAGTGCGGGTC-3'
		Reverse	5'-TCGACGAACTCCCGACGA-3'
	Unmethylated	Forward	5'-GTGTTTTATTGTGGAGTGTGGGTT-3'
		Reverse	5'-CCAATCACAAACTCCCAACAA-3'
*TWIST*	Methylated	Forward	5'-TTTCGGATGGGGTTGTTATCG-3'
		Reverse	5'-GACGAACGCGAAACGATTTC-3'
	Unmethylated	Forward	5'-TTGGATGGGGTTGTTATTGT-3'
		Reverse	5'-ACCTTCCTCCAACAAACACA-3'
*NES1*	Methylated	Forward	5'-TTCGAAGTTTATGGCGTTTC-3'
		Reverse	5'-TTATTTCCGCAATACGCGAC-3'
	Unmethylated	Forward	5'-TTGTAGAGGTGGTGTTGTTT-3'
		Reverse	5'-CACACAATAAAACAAAAAACCA-3'
*RARβ2*	Methylated	Forward	5'-GAACGCGAGCGATTCGAGT-3'
		Reverse	5'-GACCAATCCAACCGAAACG-3'
	Unmethylated	Forward	5'-GGATTGGGATGTTGAGAATGT-3'
		Reverse	5'-CAACCAATCCAACCAAAACAA-3'
*CDH1*	Methylated	Forward	5'-TTAGGTTAGAGGGTTATCGCGT-3'
		Reverse	5'-TAACTAAAAATTCACCTACCGAC-3'
	Unmethylated	Forward	5'-TAATTTTAGGTTAGAGGGTTATTGT-3'
		Reverse	5'-CACAACCAATCAACAAC ACA-3'

MSP, methylation-specific PCR.

PCR was carried out after optimizing annealing temperatures for each primer set to include 40 timed cycles of denaturation at 94°C for 30 seconds, annealing for 30 seconds, and extension at 72°C for 30 seconds. Post-MSP product analysis was performed using capillary array electrophoresis (CEQ 8000XL, Beckman Coulter, Fullerton, CA, USA), as described previously [15,24].

### Statistical analysis

Descriptive statistics were used to summarize patient characteristics and gene hypermethylation status. χ^2 ^test and Fisher's exact test were used to compare methylation status between ER-positive and ER-negative subgroups, and to compare those differences according to clinical factors, such as tumor size (T stage) and nodal status (N stage). All statistical analyses were carried out using the SAS system (SAS, Cary, NC, USA), and *P *< 0.05 was considered statistically significant.

## Results

### Methylation status of genes in ER-negative and ER-positive tumors

**Figure 1:**
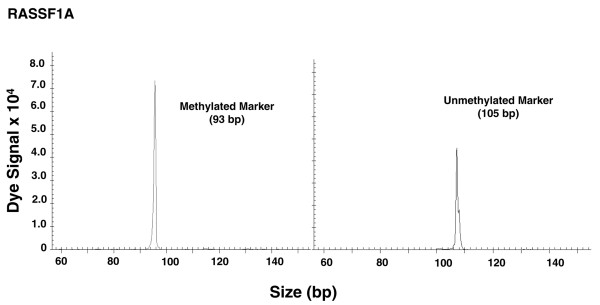
Methylated and unmethylated markers. Presented are representative methylation-specific PCR results of biomarker RASSF1A from a paraffin-embedded archival breast tissue specimens, showing methylated and unmethylated markers. bp, base pairs.

Initially, the difference in methylation status of eight genes between the age-matched and tumor background-matched ER-negative and ER-positive groups was analyzed using χ^2 ^tests. A representative example of methylated and unmethylated gene analysis from paraffin-embedded tissue is shown in Figure 1. For *RASSF1A*, *CCND2*, *GSTP1*, *TWIST*, and *APC *genes, the proportion of methylated genes was significantly higher in the ER-positive than in the ER-negative tumor group. However, no significant differences in methylation status were detected in *NES1*, *RARβ2*, and *CDH1*. Among the eight biomarkers studied, none exhibited predominance of methylation status in ER-negative tumors by univariate analysis (Table 4). Based on this finding, we conducted further analysis of the methylation status of *RASSF1A*, *CCND2*, *GSTP1*, *TWIST*, and *APC*. Second, we analyzed the differences in the methylation status of these five tumor-related genes between premenopausal and postmenopausal, T1 and T2/T3, and LN metastasis negative (N0) and positive (N1/N2) subgroups using univariate analysis. Among these five genes, there were no significant differences in methylation frequency between premenopausal and postmenopausal subgroups and T1 and T2/T3 subgroups. Only *GSTP1 *exhibited significantly more frequent methylation in the N1/N2 subgroup than in the N0 subgroup (Tables 5 to 7).

**Table 4:** Comparison of primary tumor gene methylation status relative to ER status

Gene	ER negative (*n* = 65)		ER positive (*n* = 65)		*P *value^a^
		
	*n*	%	*n*	%	
*RASSF1A*	36	55%	60	92%	<0.0001
*CCND2*	26	40%	45	69%	0.0008
*GSTP1*	4	6%	19	29%	0.0006
*TWIST*	17	26%	32	49%	0.0066
*APC*	20	31%	31	48%	0.048
*NES1*	13	20%	22	34%	NS
*RARβ2*	18	28%	17	26%	NS
*CDH1*	53	82%	54	83%	NS

^a^χ^2 ^test or Fisher's exact test was used appropriately. ER, estrogen receptor; NS, not significant.

**Table 5:** Comparison of primary tumor gene methylation status relative to patient menopausal status

Gene	Premenopause (*n* = 70)		Postmenopause (*n *= 60)		*P *value^a^
		
	*n*	%	*n*	%	
*RASSF1A*	52	74%	44	73%	NS
*CCND2*	40	57%	31	52%	NS
*GSTP1*	13	19%	10	17%	NS
*TWIST*	25	36%	24	40%	NS
*APC*	27	39%	24	40%	NS

^a^χ^2 ^test. NS, not significant.

**Table 6:** Comparison of primary tumor gene methylation status relative to T stage

Gene	T1 (*n* = 48)		T2/T3 (*n* = 82)		*P *value^a^
		
	*n*	%	*n*	%	
*RASSF1A*	34	71%	62	77%	NS
*CCND2*	30	63%	41	50%	NS
*GSTP1*	9	19%	14	17%	NS
*TWIST*	18	38%	31	38%	NS
*APC*	17	35%	34	41%	NS

^a^χ^2 ^test. NS, not significant.

**Table 7:** Comparison of primary tumor gene methylation status relative to nodal status

Gene	N0 (*n* = 76)		N1/N2 (*n* = 54)		*P *value^a^
		
	*n*	%	*n*	%	
*RASSF1A*	54	71%	42	78%	NS
*CCND2*	42	55%	29	54%	NS
*GSTP1*	8	11%	15	28%	0.011
*TWIST*	28	37%	21	39%	NS
*APC*	30	39%	21	39%	NS

^a^χ^2 ^test. NS, not significant.

### Methylation status of genes in ER-negative and ER-positive early-stage tumors

To study further the changes in tumor-related gene methylation status of both ER-positive and ER-negative breast cancers during tumor progression, we investigated the difference between both ER-positive and ER-negative primary tumors based on American Joint Committee on Cancer T and N stages. First, we compared the methylation status of five different genes in ER-negative and ER-positive tumors for T1c and T2/T3 stages (Table 8). The ER-positive group exhibited more frequent methylation of *RASSF1A *and *CCND2 *in both T1c and T2/T3 stages. *GSTP1 *and *TWIST *were more methylated in the ER-positive group in the T1c stage, but the differences did not achieve statistical significance. However, they showed significant differences in T2/T3 stage. We examined changes in the methylation frequency ratio (methylation frequency of gene in the ER-positive group/methylation frequency of gene in the ER-negative group) for each tumor-related gene during tumor progression. No difference in the methylation frequency ratio was observed between T1c and T2/T3 stages for all tumor-related genes assessed.

**Table 8:** Methylated primary ER-negative and ER-positive tumors: T1c and T2/T3 subgroups

	T1c (*n* = 48)			T2/T3 (*n* = 82)		
	
Gene	ER negative (*n* = 24)	ER positive (*n* = 24)	*P *value^a^	ER negative (*n* = 41)	ER positive (*n* = 41)	*P *value^a^
*RASSF1A*	13 (54%)	21 (88%)	0.011	23 (56%)	39 (95%)	<0.0001
*CCND2*	10 (42%)	20 (83%)	0.0029	16 (39%)	25 (61%)	0.0468
*GSTP1*	3 (13%)	6 (25%)	NS	1 (2%)	13 (32%)	0.0004
*TWIST*	6 (25%)	12 (50%)	NS	11 (27%)	20 (49%)	0.0404
*APC*	9 (38%)	8 (33%)	NS	11 (27%)	23 (56%)	0.0071

^a^χ^2 ^test or Fisher's exact test was used appropriately. ER, estrogen receptor; NS, not significant.

Next, we investigated the difference in methylation of tumor-related genes between the ER-negative and ER-positive subgroups of N0 and N1/N2 tumors (Table 9). The ER-positive group exhibited significantly (*P *< 0.05) more frequent methylation of *RASSF1A*, *CCND2*, and *GSTP1 *in both N0 and N1/N2 stages. *TWIST *and *APC *were more frequently methylated in the ER-positive group in the N0 stage, but not significantly. Similar to subgroups divided by T stage, there were no differences in methylation frequency ratio for all genes assessed between the N0 and N1/N2 subgroups.

**Table 9:** Methylated primary ER-negative and ER-positive tumors: N0 and N1/N2 subgroups

	N0 (*n* = 76)			N1/N2 (*n* = 54)		
	
Genes	ER negative (*n* = 38)	ER positive (*n* = 38)	*P *value^a^	ER negative (*n* = 27)	ER positive (*n* = 27)	*P *value^a^
*RASSF1A*	18 (47%)	36 (95%)	<0.0001	18 (67%)	24 (89%)	0.0495
*CCND2*	16 (42%)	26 (68%)	0.021	10 (37%)	19 (70%)	0.014
*GSTP1*	1 (3%)	7 (18%)	0.025	3 (11%)	12 (44%)	0.0062
*TWIST*	11 (29%)	17 (45%)	NS	6 (22%)	15 (56%)	0.012
*APC*	11 (29%)	19 (50%)	NS	9 (33%)	12 (44%)	NS

^a^χ^2 ^test or Fisher's exact test was used appropriately. ER, estrogen receptor; NS, not significant.

### Methylation status of tumor-related genes relevant to HER2/neu status

In addition to ER, we investigated differences in methylation status of the eight genes between the HER2/neu-negative and HER2/neu-positive tumor groups. First, we analyzed the relation between ER status and HER2/neu status. Using matched samples, no difference was found in the frequency of HER2/neu-positive tumors between the ER-negative and ER-positive groups. Then, the differences in methylation status of all eight genes were analyzed between the HER2/neu-positive and HER2/neu-negative tumor groups. The proportion of methylated *RASSF1A*, *GSTP1*, and *APC *genes was significantly greater in the HER2/neu-positive than in the HER2/neu-negative tumor group; no significant differences in methylation status were detected for *TWIST*, *NES1*, *RARβ2*, and *CCND2*. Among the eight biomarkers studied, *CDH1 *showed predominance of methylation status in HER2/neu-negative tumors by univariate analysis (Table 10). Next, we compared the methylation status of double-negative tumors versus breast cancers expressing either ER or HER2/neu. The double-negative breast cancer group showed less frequent methylation of *APC*, *GSTP1*, *RASSF1A*, and *TWIST *(Table 11). Logistic regression analysis revealed that both ER and HER2/neu status affect the methylation status of *APC*, *GSTP1*, and *RASSF1A *independently.

**Table 10:** Comparison of primary tumor gene methylation status relative to HER2/neu status

	HER2/neu negative (*n* = 78)		HER2/neu positive (*n* = 29)		*P *value^a^
		
Gene	*n*	%	*n*	%	
*RASSF1A*	55	71%	27	93%	0.007
*CCND2*	44	56%	15	52%	NS
*GSTP1*	10	13%	12	41%	0.0019
*TWIST*	29	37%	11	38%	NS
*APC*	26	33%	17	59%	0.0185
*NES1*	22	28%	7	24%	NS
*RARβ2*	20	26%	11	38%	NS
*CDH1*	68	87%	20	67%	0.036

^a^χ^2 ^test or Fisher's exact test was used appropriately. HER, human epidermal growth factor receptor; NS, not significant.

**Table 11:** Comparison of primary tumor gene methylation status relative to double-negative breast cancers versus cancers expressing either ER or HER2/neu

Gene	Double negative (*n* = 37)		Either ER positive or HER2/neu positive (*n* = 70)		*P *value^a^
		
	*n*	%	*n*	%	
*RASSF1A*	18	47%	64	91%	<0.0001
*CCND2*	16	43%	43	61%	NS
*GSTP1*	0	0%	22	31%	<0.0001
*TWIST*	7	19%	33	47%	0.0031
*APC*	8	22%	35	50%	0.0035
*NES1*	8	22%	21	30%	NS
*RARβ2*	10	27%	21	30%	NS
*CDH1*	33	89%	55	79%	NS

^a^χ^2 ^test or Fisher's exact test was used appropriately. ER, estrogen receptor; NS, not significant; HER, human epidermal growth factor.

### Relation between the tumor-related genes with respect to methylation status

The relation between the genes with respect to methylation status is shown in Table 12. *GSTP1 *methylation frequency is significantly related to methylation frequency of other genes. *RASSF1A *and *TWIST *methylation frequencies were significantly related to methylation frequency of three of four other genes. *CCND2 *and *APC *methylation frequency related with two of four other genes. In total, we examined 10 different relationships among five genes; seven of the 10 correlations (70%) were statistically significant (*P *< 0.05).

**Table 12:** The relation between the methylated genes relative to methylation status

Genes	TWIST	RASSF1A	CCND2	APC
*GSTP1*	<0.0001	0.008	0.0006	0.0002
*TWIST*	-	0.047	0.003	NS
*RASSF1A*	-	-	NS	0.01
*CCND2*	-	-	-	NS

^a^χ^2 ^test. NS, not significant.

## Discussion

During the past several years, new molecular biomarkers have been discovered that are important targets for the diagnosis and therapy of breast cancer [3,42]. ER and HER2/neu are important prognostic biomarkers and therapeutic targets in primary breast cancer. ER-negative tumors appear to be more malignant [4,7,8,43], resulting in a poorer prognosis than with ER-positive tumors [5,9,44]. The present study was conducted to identify differences in epigenetic events related to ER expression by infiltrating breast cancer. To date, few studies have rigorously assessed matched paired ER-negative and ER-positive primary breast tumors for epigenetic differences. We focused on the epigenetic differences between ER-positive and ER-negative breast cancers, and used tumor specimen pairs matched for patient age, size, nodal status, and presence or absence of distant metastases. This sampling enabled rigorous analysis, and the results imply that epigenetic features of ER-positive tumors are different from those of ER-negative tumors.

Widschwendter and coworkers [3] demonstrated that methylation of *APC *correlated with ER positivity. Our data are consistent with this previous report. Furthermore, we demonstrated a significant difference in methylation status of *RASSF1A*, *CCND2*, *GSTP1*, *TWIST*, and *APC *between the ER-positive and ER-negative groups. In contrast, Li and colleagues [45] reported that ER-positive patients exhibited a higher frequency of *TWIST *methylation and a lower frequency of *CDH1 *methylation than did ER-negative patients. They also found no significant differences in the methylation frequencies of *RARβ2*, *CCND2*, and *CDH1 *between ER-positive and ER-negative groups. The reason for the dissimilarity in study findings may be due to differences in methylation analysis; Li and colleagues assessed methylated PCR products by gel electrophoresis, which is more subjective and less sensitive than capillary array electrophoresis analysis of methylated PCR products. The discrepancy may also have resulted from differences in the approach to the particular specimens assessed. Because methylation and ER status change with tumor progression [25], care should be taken when sampling ER-positive and ER-negative tumors to evaluate epigenetic changes and clinical associations.

To clarify when differences in methylation status between ER-positive and -negative tumors occur, we compared differences in methylation status between T1c and T2 stage subgroups. The ER-positive group exhibited significantly more frequent hypermethylation of two genes (*RASSF1A *and *CCND2*), independent of T stage. Moreover, the ratio of methylation frequency does not differ between T1c and T2/T3 stage subgroups. This observation indicates that the differences in methylation patterns do not significantly change when breast tumors progress from T1c to T2/T3 stage. Similarly, no difference in the methylation frequency ratio was detected between LN metastasis negative and positive tumors. Previously, we reported that *GSTP1 *hypermethylation correlates with LN metastasis [24]. Similarly in this study, only GSTP1 hypermethylation was found to be more frequent in the LN metastasis positive group than in the negative group. In both LN metastasis positive and negative groups, *GSTP1 *hypermethylation was found to be more common in ER-positive than in ER-negative tumors. Furthermore, the differences in methylation status of *RASSF1A *and *CCND2 *between the ER-positive and ER-negative groups can be recognized in early stages of cancer, such as the T1c or N0 stages. These findings suggest that ER expression may influence epigenetic changes in early stages of breast cancer.

HER2/neu is another important prognostic marker for breast cancer. HER2/neu gene over-expression is identified in 15% to 25% of invasive breast carcinomas, and is related to metastatic potential and poor survival [35,46]. In our study, HER2/neu over-expression was identified in 27% of breast cancers, and was independent of ER status. A negative relation between ER status and HER2/neu over-expression has been documented by others [9,38,39]. One of the plausible explanations for the lack of difference found in the frequency of HER2/neu-positive tumors between the ER-negative and ER-positive groups is that patients included in the present study were relatively young (average age 51 years). According to Huang and coworkers [38], in women younger than 45 years the inverse association between ER and HER2/neu was not apparent. Of our sample population, 37% of patients were under 45 years old, and this age distribution may explain why we could not detect an inverse relation between ER and HER2/neu status. Regarding gene methylation and HER2/neu status, Fiegl and colleagues [47] reported that methylation of *CDH13*, *MYOD1*, *PGR*, and *HSD17B4 *exhibited a positive association with HER2/neu expression. We demonstrated that *APC*, *GSTP1*, and *RASSF1A *are more frequently methylated in the HER2/neu over-expressed group. These findings indicate that there are epigenetic differences between HER2/neu breast tumors, and logistic regression analysis showed that these differences are independent of ER status.

Clinically, HER2/neu-negative, ER-negative, and PR-negative breast cancers are referred to as triple-negative breast cancers. About 85% of triple-negative breast tumors phenotypes are deemed to be basal-like subtypes, and they are associated with poor clinical outcome [48]. Because the progesterone receptor status of our samples was not well defined, we compared the methylation status of ER-negative and HER2/neu-negative breast tumors (double-negative) with breast cancers expressing either ER or HER2/neu. Double-negative breast tumors exhibited frequent hypermethylation in *APC*, *GSTP1*, *RASSF1A*, and *TWIST*, revealing the presence of epigenetic differences between double-negative breast tumors and breast cancers expressing either HER2/neu or ER. Furthermore, both ER and HER2/neu status contributed independently to the difference in expression of *APC*, *GSTP1*, and *RASSF1A*. There is evidence that gene expression heterogeneity occurs in basal-like tumors of triple negatives; these subgroups are present with different pathology and clinical properties. Triple negative and ER and HER2/neu-positive status and gene expression levels of tumors may be quite heterogeneous in tumor populations, thus contributing to different subclassifications. Other additional biomarkers may be needed to subclassify phenotypes better [49,50].

Another interesting finding is the correlation of methylation status between the tumor-related genes. Methylation status of *RASSF1A*, *CCND2*, *GSTP1*, *TWIST*, and *APC*, which was significantly higher in the ER-positive group, was correlated with the methylation status of two or more of the four other genes. Nonrandom distribution of methylation in tumor-related genes has previously been reported; Nass and coworkers [51] and Li and colleagues [45] found coincident methylation of *CDH1 *and *ESR1*, and Parrella and coworkers [52] reported that *ESR1 *promoter hypermethylation status correlates with those of *CDH1*, *GSTP1*, *CCND2*, and *TRb1*. Our findings revealed that occurrence of promoter region hypermethylation of *RASSF1A*, *CCND2*, *GSPT1*, *TWIST*, and *APC *are associated, whereas *CDH1 *methylation exhibited no correlation with methylation of those five genes (data not shown). This apparent nonrandom distribution of promoter hypermethylation of some genes suggests the existence of specific factors causing selective promoter region hypermethylation of tumor-related genes.

## Conclusion

In this study, we demonstrated – for the first time – that epigenetic differences between ER-positive and ER-negative breast tumors arise early in cancer development and persist during cancer progression. Furthermore, we reported epigenetic differences between HER2/neu-positive and HER2/neu-negative breast tumors, and between double-negative breast tumors and breast tumors expressing either HER2/neu or ER. Taking advantage of potential reversibility of DNA methylation, epigenetic therapies directed against various cancers have been in development; 5-azacytidine, 5-aza-2'-deoxycytidines, procainamide, and hydralazine are promising agents. Investigators are attempting to combine epigenetic therapy with other standard therapies [2,53]. For breast cancer, one approach may to combine hormone therapy and antimethylation treatment. Integrated information regarding clinical factors influencing therapeutic principles and epigenetic features, such as promoter hypermethylation of tumor-related genes, will be important for combining epigenetics targeting drugs and standard chemotherapy.

## Abbreviations

APC, adenomatous polyposis coli; CCND2, cyclin D2_; _CDH1, E-cadherin; ER, estrogen receptor; GSTP1, glutathione S-transferase P1; HER2/neu, human epidermal growth factor receptor 2; LN, lymph node; MSP, methylation-specific PCR; NES1, normal epithelial cell-specific 1 or kallikrein 10; RARβ2, retinoic acid receptor- β2; RASSF1A, RAS association domain family 1A; SLN, sentinel lymph node; TWIST, human basic helix-loop-helix DNA binding protein.

## Competing interests

The authors have submitted a patent application relating to the content of this manuscript.

## Authors' contributions

ES and MS performed methylation assays for the studies. M-SS was the biostatistician in the study. SLN performed assays and assembled the data. A-TV helped with assays. AEG performed the clinical review of the patients and reviewed the study design. DSNH was the principle investigator and designed and reviewed the study.
